# Impact of liquid dosing and associated cell ‘drowning’ in a 3D human bronchial epithelial model used for protease exposure studies

**DOI:** 10.3389/ftox.2026.1781231

**Published:** 2026-05-27

**Authors:** Georgina Hopkins, David Sheffield, Hugh Barlow, Anja Lalljie, Stella Cochrane, Lucy C Fairclough

**Affiliations:** 1 School of Life Sciences, The University of Nottingham, Nottingham, United Kingdom; 2 SERS, Unilever, Colworth Science Park, Sharnbrook, United Kingdom

**Keywords:** ALI (air liquid interface), bronchial epithelial cells, extracellular vescicles (EVs), MPPD model, protease

## Abstract

**Introduction:**

As part of a programme of work developing human-relevant New Approach Methodologies (NAMs) for next-generation risk assessment (NGRA), use of a human air-liquid interface (ALI) bronchial epithelial model (MucilAir™) to investigate the impact of exposure to protease was assessed.

**Methods:**

To guide *in vitro* dosing, scenarios representing low-high risk of sensitisation after 8 h of protease exposure in an occupational setting were modelled by performing simulations with the Multiple Path Particle Dosimetry (MPPD) model with refinement of the outputs using a tracheobronchial/alveolar clearance model, to estimate tracheobronchial tissue doses. With no effects seen at these concentrations, higher concentrations were subsequently tested to identify thresholds for biological effects. Repeated apical liquid exposures over 8 h were applied using a wide concentration range of protease (0.00125–7812 μg protease protein/mL) or phosphate-buffered saline (PBS) control. Effects on epithelial barrier integrity, cytokine production, and extracellular vesicle (EV) dynamics were measured.

**Results:**

Reduced transepithelial electrical resistance (TEER), increased EV and IL-8 secretion were observed using a PBS control reflecting the impact of liquid dosing alone. No significant protease-related adverse effects were observed at concentrations of 0.5 μg protease protein/mL or lower when compared to the PBS control. At concentrations of 75 μg protease protein/mL or more, however, TEER was significantly reduced and mucin and tetraspanin expression on EVs was degraded.

**Discussion:**

Here, we show the impact of liquid dosing when investigating the effects of materials on bronchial epithelia, and challenges encountered when working with proteases. This work provides a foundation for developing *in vitro* methodology for generation of data for use in risk assessment of inhalation of enzymes and other materials. It is hypothesised that nebulised protease delivery could be a more suitable alternative and better replicate *in vivo* (human) inhalation dynamics.

## Introduction

The airway epithelium serves as a critical barrier that protects the respiratory tract from foreign substances. It is selectively permeable due to a complex network of receptors, transporters, and tight junctions, which regulate the movement of substances across the epithelium (van Ree et al., 2014). Exposure of the epithelial barrier to respiratory irritants can result in the degradation of the tight junctions and subsequent secretion of inflammatory cytokines (Noureddine et al., 2022). In addition, extracellular vesicles (EVs), which are membrane-bound vesicles released from all cells, have also been shown to play a critical role in promoting Th2 and Th17 immune responses after airway epithelial exposure to allergens (Ambrożej et al., 2022a; Ax et al., 2020; Browne et al., 2025; Tucis et al., 2024). Importantly, a defective epithelial barrier has been demonstrated in many inflammatory diseases, such as allergies and autoimmune disorders (Akdis, 2021). This has led to a hypothesis being proposed that the increase in immune-mediated diseases such as allergies that has occurred in recent years is due, at least in part, to damaged epithelial barriers - the “epithelial barrier hypothesis” (Akdis, 2021).

Inhalation exposure to enzymes, such as proteases, is one of many factors postulated to play a role in epithelial barrier disruption and development of IgE-mediated allergies. This can occur in many contexts, for example, because of exposure to environmental allergens (e.g., the house dust mite allergen Der p1) or in occupational settings where enzymes are handled (such as detergent manufacturing) without adequate controls. Protease enzymes are naturally derived from animals, plants, and microbes, constituting the largest product segment in the global industrial market for enzymes, primarily due to their extensive use in household cleaning products (Song et al., 2023).

Although the epithelial barrier hypothesis was introduced in the late 2010 s, it has been known for decades that inhalation exposure to enzymes of bacterial and fungal origin, such as those used for many decades in cleaning and detergent products, have the potential to induce allergic sensitisation of the respiratory tract and occupational respiratory allergy/asthma (Basketter et al., 2010; Sarlo, 2003; Adisesh et al., 2011; Flindt, 1969). Reflecting this knowledge, years of work has been undertaken to identify exposures of concern and implement occupational hygiene measures to manage this risk, resulting in avoidance of occupational allergic disease (Sarlo, 2003; Basketter et al., 2021; Basketter et al., 2015). Additionally, a thorough risk assessment of the use of enzymes in consumer products ensures this potential risk is also managed and no health concerns have been identified through use of such products when manufacturers follow best practice guidance, such as that published by the ACI/AISE (AESI, 2025), and as evidenced by recently published data (David et al., 2024).

In the field of toxicology, steps are being taken toward more mechanism-focused and human-relevant approaches to risk assessment, driving the development of new methods, including *in vitro* techniques, incorporating those for assessment of effects of materials on the human respiratory and immune systems (Cochrane et al., 2024; de Ávila et al., 2025; Dent et al., 2018; Dent et al., 2021; Naidenko et al., 2021; Wang et al., 2022). Most existing *in vitro* non-animal methods describe the use of ‘epithelial’ cell lines, such as BEAS2B, Calu-3, and HaCaT (Oh et al., 2022; Lujan et al., 2019), where gene expression and biological function in these single cell lines can differ from that of intact human airway epithelium. Furthermore, traditional submerged cell culture models fail to mimic the differentiated structure and function of the airway epithelium (Allouche et al., 2025). Therefore, organotypic multi-cell type models grown at the air-liquid interface (ALI) for the human airway have been developed, which allow for the development of pseudostratified epithelium which closely mimics *in vivo* conditions. One such model is the MucilAir™ model, which consists of primary ciliated cells, mucus-secreting goblet cells and basal cells, and has been demonstrated to reflect the physiological conditions in the upper airways (Balharry et al., 2008; Welch et al., 2021). It is worth highlighting that this model addresses the epithelial contribution to airway responses, rather than the full complexity of immune-mediated processes.

This study investigated the potential use of the MucilAir™ model to provide a robust, *in vitro* assessment of the impact of protease enzyme on human upper airway epithelia. Additionally, to understand how *in vitro* dosing and effects relate to inhalable concentrations, including those spanning occupational exposures associated with low and high risks of sensitisation, modelling (including the use of the MPPD model) was undertaken to extrapolate these exposures. As such, a wide tissue dosing range was implemented to account for any associated uncertainties and understand concentrations at which significant effects occur *in vitro*.

Without a nebuliser system to aerosolise protease, and reflective of the approach taken by many research groups, an apical liquid exposure approach was utilised, re-exposing epithelial cells every 2 h and subsequently removing residual solution each time over 8 h, to simulate repeated protease exposure, as might occur in an occupational setting. MucilAir™ cells and supernatants were then analysed for epithelial barrier function, cytokine production, and EV characterisation.

## Materials and methods

### Exposure dose selection

A range of airborne enzyme protein concentrations covering those associated with a low to high risk of allergic sensitisation in an occupational setting (1–10,000 ng protease per m^3^) were extrapolated to bronchial tissue doses using the MPPD model (v3.04 from ARA) with refinement of the outputs for the tracheobronchial region using a tracheobronchial/alveolar clearance model developed as described in de Avila et al., 2025 (de Ávila et al., 2025). The resulting tissue doses identified were used for setting an initial dosing range, recognising they would have a high degree of associated uncertainty. For further details on the modelling methodology, including MPPD parameters used, and initial dosing concentrations identified see Supplementary Material S1. No significant protease effects were observed across the lowest concentration ranges tested. Subsequently, these were increased to identify concentrations at which significant treatment-related effects occurred. Table 1 shows the 10 exposure doses tested in this study, and their equivalent concentrations in terms of surface loading. Data is provided for the latest protease concentration ranges tested spanning those with no significant protease-related effects *in vitro* to those resulting in significant changes.

**TABLE 1:** The liquid exposure concentrations used in this study and equivalent inhaled surface loading concentrations using 0.33 cm^2^ cell inserts.

Apical liquid exposure (µg/mL)	Equivalent surface loading (µg/cm^2^)
0.0013	0.0002
0.0313	0.0049
0.1000	0.0156
0.5000	0.0781
2.5000	0.3906
12.500	1.9531
75.000	11.719
150.00	23.438
312.00	48.750
7,812.0	1,220.6

### Donor characteristics and exposure regime

MucilAir^TM^-bronchial epithelial samples from seven young, male, healthy, non-smoker donors were utilised in this study. The characteristics and protease concentrations utilised for these donors are shown in Table 2. The TEER and cilia beating frequency measurements were obtained from the supplier before shipping the cells. The donors were ordered sequentially, and so the protease concentration range reflects the dynamic selection process described above.

**TABLE 2:** Donor Characteristics and exposure regime of MucilAir^TM^-bronchial epithelial samples.

Donor	Age	Sex	Origin	TEER before arrival in lab (Ω.cm2)	Cilia beating frequency (Hz) before arrival in lab	Time from ALI formation to experiment (Days)	Protease range (µg protein/mL)
MD0895	21	Male	Caucasian	273±6	10.5±0.2	50	0 (PBS), 0.00125, 0.03125, 0.5
MD0937a	29	Male	Unknown	368±9	9.6±0.2	64	0 (PBS), 0.00125, 0.03125, 0.5
MD571a	35	Male	Unknown	769±12	8.9±0.2	53	0 (PBS), 0.00125, 0.03125, 0.5
MD0858a	21	Male	Caucasian	388±15	8.7±0.2	51	0 (PBS), 0.1, 0.5, 2.5, 12.5
MD0571b	35	Male	Unknown	865±10	8.5±0.2	67	0 (PBS), 0.5, 12.5, 150, 312
MD0858b	21	Male	Caucasian	346±9	8.1±0.4	78	0 (PBS), 12.5, 75, 150, 312
MD0937b	29	Male	Unknown	392±13	10±0.2	43	0 (PBS), 0.5, 12.5, 312, 7,812

### Culture of primary bronchial epithelial cells

The MucilAir^TM^-bronchial (Cat no. EP01MD) samples were purchased from Epithelix Sárl, and each were a fully differentiated bronchial epithelial model reconstituted from primary human cells taken from the donors described. The samples arrived in the format of 24-well transwell inserts (0.33 cm^2^), which were then placed in a sterile 24-well plate with 700 µL of phenol red-free MucilAir™ culture medium (Cat no. EP06MM, Epithelix Sárl) placed in the basolateral compartment, and then transferred to a humidified incubator (37 °C; 5% CO_2_). The culture medium was changed every 2–3 days.

### Apical liquid exposure approach

An apical wash with phosphate-buffered saline (PBS) was performed on the MucilAir™ insert 4 days prior to any exposure experiment, as suggested by the manufacturer, to mimic a normal amount of mucus build-up.

The protease solution (cat no. P3111, Merck), which is a serine protease secreted by a *Bacillus* sp. (Savinase® 16.0L), was first analysed by bicinchoninic acid (BCA) assay to determine the protein concentration (See methods section ‘BCA Assay’). The stock solution was then diluted to the desired range of concentrations in sterile PBS (Cat no. D8527, Merck) and stored at 4 °C. Solutions were brought to room temperature before each 2 h exposure, and replaced in the fridge. In the certificate of analysis, the proteolytic activity of the stock protease solution was given as 17 KNPU/G. A random order generator (URL: https://www.random.org/lists/) was used to determine the plate layout for each experiment. Triplicate wells were utilised for each protease concentration exposure.

Aliquots (50 μL) of each protease solution was applied apically to the epithelial transwell insert four times over an 8-h period and replaced in the incubator at 37 °C; 5% CO_2_ each time. This was to mimic a prolonged enzyme exposure scenario. PBS without enzyme was utilised as a control treatment. Before each application, any residual enzyme or control PBS solution from the previous application was removed. After 8 h, the final enzyme or PBS solutions were removed from the apical surface, and the samples were incubated at 37 °C; 5% CO_2_ for a further 16 h.

**FIGURE 1:**
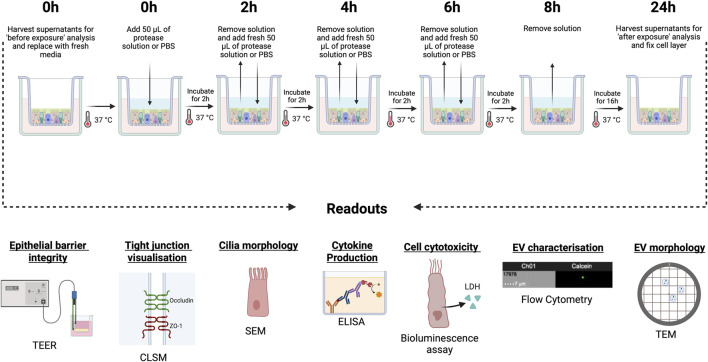
Apical Liquid Exposure Experimental outline. For treated cells, commercially supplied protease is diluted to the desired concentrations and applied to the surface (apically) of healthy human bronchial epithelial cells, gradually over 8 h to mimic prolonged exposure. For control cells, the same dosing protocol was used with sterile-filtered PBS applied rather than an enzyme solution. 24 h after the first dose of protease or for controls PBS, the epithelial cells and their supernatants were then harvested and analysed for epithelial barrier integrity and immune responses. TEER measurements, SEM and confocal microscopy were utilised to measure epithelial barrier integrity, cilia morphology, and tight junction integrity. ELISAs were conducted to measure cytokine responses. A bioluminescence assay for LDH quantification was used to assess cell viability, and EVs were characterised by flow cytometry (ImageStream), and TEM for morphology. Created using BioRender.

An overview of this apical liquid exposure methodology is presented in Figure 1, along with details of measurements taken, which are described further in the following sections.

### BCA assay

Protein concentrations of the stock protease solution were measured using a BCA protein assay kit (ThermoFisher, Loughborough, United Kingdom) according to manufacturer’s instructions. Briefly, the contents of one Albumin Standard (BSA) ampule were diluted into vials, using PBS as the diluent, at final BSA concentrations of 2000, 1,500, 1,000, 750, 500, 250, 125, 25 and 0 μg/mL. The BCA working reagent was prepared by mixing 50 parts of BCA reagent A with one part of BCA reagent B (50:1, Reagent A:B) and was then added to standards and sample replicates in a 96-well plate and mixing on a plate shaker for 30 s. The plate was then incubated at 37 °C for 30 min. The absorbance was then measured near 562 nm on a GloMax Explorer plate reader (Promega). A standard curve was made by plotting the 562 nm measurement for each BSA *versus* its concentration in μg/mL and used to determine the protein concentration of serially diluted protease stock solution.

### Protease activity test

An EnzCheck protease activity assay kit (Cat no. E6638, Invitrogen) was purchased to quantify the protease activity of protease solutions when measuring enzyme storage conditions, as well as to quantify protease activity in apical washes between 2 h exposures, and finally to quantify activity after the addition of 0.2 mM of Phenylmethylsulfonyl fluoride (PMSF) for 1 h on a shaker (Merck, cat no. 93482).

For the enzyme storage testing, the protease solution was diluted to 100, 500, 2,500, and 12,500 ng protein/mL in PBS and stored at room temperature or at 4 °C in the fridge. After 8 h, aliquots of the samples were taken and 100 µL was added to a 96-well plate. Aliquots from apical washes in between 2 h protease exposures were also added to a 96-well plate. And finally, aliquots of protease solutions incubated with 0.2 mM PMSF were added to a 96-well plate.

All samples were then incubated with an equal amount of Boron-Dipyrromethene (BODIPY) FL casein for 1 hour, protected from light, at room temperature. The fluorescence was read in a GloMax Discover Microplate Reader (Promega, US) to measure casein digestion by the enzyme solutions, at emission 500–550 nm, excitation 475 nm. The limit of detection for the assay was calculated by blank reading + 3xSD of the blank reading.

### Monocyte activation test

Human peripheral blood mononuclear cells (PBMCs) from four donors were thawed and pooled together (Ethical approval by The University of Nottingham’s Medical School Ethics Committee (Ref. 232–1902)). The PBMCs were cultured in Roswell Park Memorial Institute (RPMI)-1,640 + 5% human AB serum at 4 × 10^5^ cells/mL. Protease solution from *bacillus* sp. was serially diluted and incubated with the PBMCs for 20 h at 37 °C; 5% CO_2_. A serial dilution of Endotoxin USP (Cat no. 1235503, Merck) was also incubated with the PBMCs as a positive control. After 20 h, an IL-6 ELISA was performed (See cytokine ELISA methods section for ELISA protocol).

### TEER measurement

To determine the integrity of the epithelial barrier, transepithelial electrical resistance (TEER) measurements were conducted before enzyme exposure, and 24 h after first dose of protease. Cell Inserts were transferred into wells containing 750 µL of room temperature PBS and an aliquot of 300 µL PBS was also added to each apical chamber. TEER was then measured using a Millicell-ERS voltohmeter with chopstick electrodes (Cat no. MERS00002, Merck). The voltohmeter was calibrated before each experiment, using a STX04 test electrode. Values were adjusted for the resistance of the MucilAir™ support membrane (corrected value = measured value − blank insert value), then corrected for the tissue surface area (0.33 cm^2^).

### Immunofluorescence staining

After supernatants were harvested, the epithelial cells within transwells were washed four times with PBS; 200 µL apically and 750 µL basally. Cells were then fixed with 4% paraformaldehyde (Cat no. 420801, Biolegend), 200 µL applied apically and 750 µL basally, for 30 min at room temperature. The fixation buffer was then washed off with PBS and the cells were stored in 1 mL of PBS at 4 °C for up to 1 week. The samples were then permeabilised with 200 µL into apical and 750 µL into basal compartments of 0.05% Triton-X in PBS for 30 min at 4 °C. The cells were washed with PBS three times and then blocked for 1.5 h with 100 µL of immunofluorescence buffer (PBS containing 5% BSA (Cat no. A7284, Merck)), 0.2% Triton-X (Cat no. T8532, Merck), and 0.05% Tween20 (Cat no. P1379, Merck)) in the apical chamber. The blocking buffer was removed and cells were incubated with 50 µL of 5 μg/mL AF488–labelled anti-occludin mAb (Cat no. 10073504, Invitrogen) and 5 μg/mL anti-zonula occludin 1 (ZO-1)–AF647 mAB (Cat no. 15648028, Invitrogen), applied apically, overnight at 4 °C in the dark. The cells were then washed with immunofluorescence buffer and removed from the transwell with a scalpel. The cells were then stored in a 24 well plate with 1 mL PBS, at 4 °C protected from light for up to 1 week. Specimens were examined under a Zeiss 900 CLSM confocal microscope.

### Scanning electron microscopy

A separate batch of inserts were fixed with 4% paraformaldehyde (Cat no. 420801, Biolegend), 200 µL applied apically and 750 µL basally, for 30 min at room temperature. The fixation buffer was then washed off with PBS and the cells were stored in 1 mL of PBS at 4 °C for up to 1 week. The PBS was then removed and inserts were dehydrated by passing through a graded series of 1 mL ethanol (Cat no. 153386F, VWR) dilutions (50, 70, 90, and 100% in ultrapure water). Dehydrated samples were then dried using 50 µL of hexamethyldisilazane until evaporated, and then mounted on an aluminium stub and sputter coated with platinum (Cat no. AGB7341, Agar Scientific), before viewing on a JEOL IT-200 scanning electron microscope (SEM).

### Cytokine ELISAs

Basal MucilAir™ cell supernatants were centrifuged at 300 *g* for 8 min to remove any cells. The supernatant was then stored at −80 °C until use in ELISAs. ELISA kits for the detection of IL-6 and IL-8 were purchased from R&D Systems (Cat no. DY206 and DY208, respectively). All ELISAs were performed according to the manufacturer’s instructions. Briefly, a 384-well plate was coated overnight at room temperature with 20 μL of capture antibody. The plate was then washed with 0.05% Tween® 20 in PBS, three times. The plate was subsequently blocked with PBS containing 1% BSA at room temperature for 1 h. The plate was then washed as before. The supernatant was thawed and used neat for IL-6 ELISAs, or one in 20 for IL-8 ELISAs, before adding 20 μL of diluted samples to the wells, as well as 20 μL of standards. The plate was incubated for 2 h at room temperature to allow cytokine binding the capture antibody. The plate was washed again before adding 20 μL of detection antibody for 2 h at room temperature. The plate was subsequently washed and streptavidin-HRP was added to each well for 20 min at room temperature, in the dark. The plate was washed again before adding 50 μL of TMB (Cat no. 34029, Thermo Fisher Scientific) for 20 min at room temperature, in the dark. Finally, 50 μL of 2NH_2_SO_4_ was added to stop the reaction, and the absorbance of the plate was read at 450 nm a GloMax Explorer plate reader (Promega). Results were analysed in GraphPad Prism Software. The limit of detection for each ELISA run was calculated by blank reading + 3xSD of the blank reading.

### LDH release assay

A cell viability assay (LDH-Glo Cytotoxicity Assay, Promega) was performed to determine possible cell toxicity. Basal supernatants were stored in 1:10 in LDH buffer (200 mM Tris-HCl (pH 7.3), 10% glycerol, 1% BSA) at −20 °C until use. Samples were then thawed and the assay performed according to the manufacturer’s instructions. Briefly, 20 μL of sample was added in duplicate to a white opaque 384-well assay plate and mixed with 20 μL of detection reagent. The plate was incubated for 1 h at room temperature before reading luminescence on a GloMax Explorer plate reader (Promega). To calculate percentage cytotoxicity, a 10% TritonX-100 treatment was added to epithelial cells as a positive control for LDH release. The following calculation was then performed:

$$\text{Percent cytotoxicity}=100\;x\;\frac{\left(\text{Experimental}\;\text{release}-\text{medium}\;\text{background}\right)}{\left(\text{Triton}-X\;\text{release}-\text{medium}\;\text{background}\right)}$$

### Transmission electron microscopy

MucilAir™ EVs were isolated by size exclusion chromatography and placed on carbon-coated 300 Cu mesh transmission electron microscopy (TEM) grids (company) for 15 min and then fixed with 2% paraformaldehyde for 10 min. The grids were washed 5 times with deionised water before incubating with 4% Uranyl Acetate for 10 min. Excess stain was removed before allowing to dry completely, and then grids were imaged using a Tecnai BioTwin TEM, operating at 80 kV, and images were recorded on a Gatan Orius camera.

### Characterisation of EVs by flow cytometry

Supernatants were removed from transwells 24 h after the first protease dose and centrifuged at 300 g for 8 min to remove any cells. Aliquots (40 μL) of supernatants were then immediately incubated with 10 μM calcein AM (Cat no. 425201, Biolegend), 2.5 μg/mL of tetraspanins CD9 APC, CD63 PE, and CD81 PEVio615 (Miltenyi) for 1 h at 37 °C to stain EVs. The samples were then diluted to 200 μL in filtered PBS and run on the ImageStreamX MkII (Amnis).

### Sizing of EVs

The ImageStream X MkII, was used to run a vFC™ EV analysis assay kit (Cellarcus Biosciences) to determine EV size, and performed according to the manufacturer’s instructions. Briefly, the instrument was first calibrated using vCal™ nanoRainbow beads and the manufacturer’s provided IDEAS layout. A synthetic vesicle size standard (5 µL), Lipo100™ or 5 µL of primary epithelial cell supernatant, were then stained with a membrane stain, vFRed™, at 1X concentration in vFC™ staining buffer, in a total volume of 50 µL for 1 h at room temperature, in the dark. Samples were then diluted 1:30 in staining buffer before running on the ImageStream X MkII. Files were imported into IDEAS software and exported as FCS files, before analysing in FCS Express (*DeNovo* software) using the manufacturer’s provided layouts. Using the Lip100™ size standard, with a size range of 80–140 nm as determined by NTA, vFRed-positive events were selected and calibrated using FCS Express transformations tool and inputting a surface area equation generated by the manufacturer’s layout. This size calibration was then applied to samples from primary epithelial cells to size the EVs.

### Statistical analysis

All statistical analyses were performed using GraphPad Prism v 10.4.1. Data are presented as mean ± standard deviation (SD) unless otherwise stated. The number of biological replicates (independent inserts) for each donor are specified in the corresponding figure legends.

Data were first assessed for normality using the Shapiro-Wilk test. Where data met assumptions of normality and homogeneity of variance, parametric tests were applied. Specifically, one-way analysis of variance (ANOVA) was used to compare treatment groups, followed by Dunnett’s post hoc test to compare each protease exposure condition to the PBS control. Where data did not meet parametric assumptions, the non-parametric Kruskal–Wallis test was applied, followed by Dunn’s multiple comparisons test. For EV data, a mixed-effects model with Dunnett’s multiple comparisons testing was used to assess the effects of protease exposures on each tetraspanin marker, compared to the PBS control. See figure legends for specific statistical test for each readout.

Flow cytometry data collected using the ImageStream X MkII was analysed using IDEAS 6.2 software and subsequently FCS Express 7.24 (*DeNovo*) for sizing. The data were then imported into Prism software, version 10.4.1 (GraphPad), for statistical analysis.

For the statistical modelling of TEER data, each measurement from the stimulated samples was compared against the threshold determined from the baseline. Thresholds were set at P25–1.5 x IQR (a standard way of determining values that are below expected levels). A logistic regression model was fit with log concentration as covariate with “success” occurring when the value was within the threshold value and “failure” when it was more extreme. Models were fit using the Logistic Procedure in SAS 9.4. The concentration at which there is a certain probability that the response will be below the threshold is determined from this model fit.

## Results

### ‘Drowning’ effect after apical liquid exposures

**FIGURE 2:**
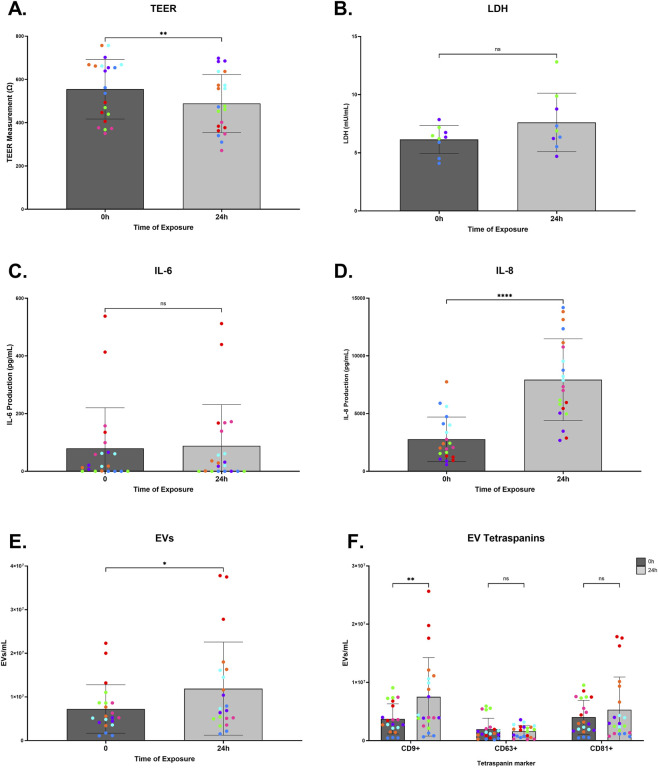
The effect of liquid dosing with PBS on MucilAir^TM^ readouts. MucilAir™ bronchial epithelial inserts were exposed to an apical liquid dose of PBS over 8 h, before measuring the following readouts at 24 h: **(A)** TEER, **(B)** LDH production, **(C)** IL-6 production, **(D)** IL-8 production, **(E)** EV production, and **(F)** the number of EVs expressing CD9, CD63, or CD81 tetraspanins. A Wilcoxon matched-pairs signed rank test was conducted for ‘A-E’, and a two-way ANOVA with Sidak’s multiple comparisons was conducted for ‘F’. N = 7 donors, except for ‘B’ which has three donors, with three replicate inserts measured for each exposure. Each donor is colour coded.

The data presented in Figure 2 are the readouts at 0 h and 24 h after apical liquid exposure to the PBS control only. This data highlights the impact on MucilAir™ of a liquid dosing approach. There was a significant decrease in epithelial barrier integrity, as indicated by a decrease in TEER measurement (p = 0.0041) (Figure 2A), but no changes in LDH production which suggests cell viability was not affected (Figure 2B). Cytokine analysis highlighted no differences in IL-6 production after PBS exposures (Figure 2C), but IL-8 production did significantly increase (p < 0.0001), indicating stimulation of the cells due to liquid dosing (Figure 2D). The number of EVs produced by the epithelial cells also significantly increased (p = 0.0175) (Figure 2E), with an increased surface expression of CD9 (p = 0.0045), but not CD63 or CD81 expression (Figure 2F). Due to these effects on MucilAir™ cells and reduced barrier integrity by the repeated liquid dosing strategy, the protease exposure results sections in this manuscript are presented as the percentage change in a readout from 0 h to 24 h of exposure. The percentage change value was then statistically compared to the percentage change of the PBS control, to account for these background effects.

### Protease characterisation

**FIGURE 3:**
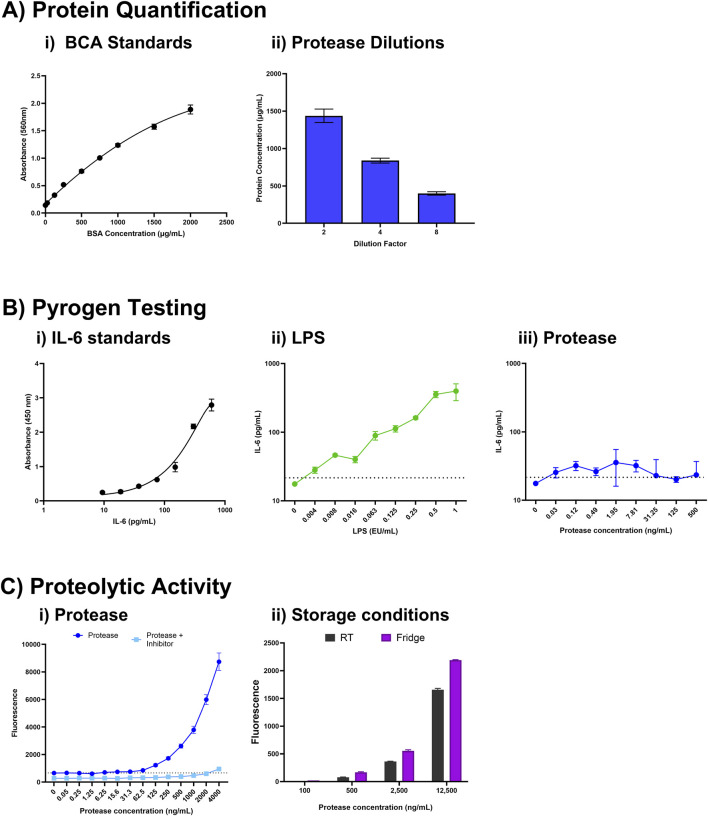
Protease Characterisation. **(A)** The protease solution was quantified for their protein content by BCA. (I) Using the absorbance (450 nm) of BSA standards to interpolate results, (ii) the protease solution was diluted by factors of 2, four and eight and their protein concentration measured (N = 3). **(B)** Pyrogen testing of protease solution using monocyte activation test. (I) IL-6 standard curve to interpolate results, (ii) IL-6 production from LPS dilutions, (iii) IL-6 production from protease dilutions (N = 4). **(C)** (I) Proteolytic activity of protease solution by casein digestion assay, with and without the presence of protease inhibitor cocktail (N = 3). (ii) Proteolytic activity of diluted protease solutions at RT and at 4 °C for 8 h (N = 2). Horizontal dotted line represents the limit of detection (LOD) of the assay. Vertical error bars represent the standard deviation.

Before exposure experiments were conducted, it was essential to characterise the protease solution for its protein content, pyrogen contamination, and proteolytic activity. First, the protein content of the protease solution was established. The enzyme solution was serially diluted in PBS and a BCA assay performed. Using the BCA standards to interpolate results, the average protein content was 122.58 mg/mL (Figure 3A). This value was subsequently used to perform all protease dilution calculations for the exposure experiments.

Next, to ensure any effects on the epithelial cells are because of the protease exposures and not any contaminating pyrogens, a monocyte activation test was carried out on the protease stock solution. LPS was used as a positive control. Figure 3B indicates that the IL-6 ELISA detected minimal pyrogen levels in the protease solution. Using the LPS curve to interpolate values, the pyrogen levels were calculated to be below 0.004 EU/mL in the protease solution.

An enzymatic activity level was provided on the certificate of analysis for the protease stock solution, 17 KNPU/G, which would equate to ∼0.14 KNPU per mg of protein based on a protein content of 122.58 ng/mL. To monitor activity over time and impact storage conditions, etc., the enzymatic activity of the protease solution was also quantified using a casein digestion assay. Using this assay, the limit of detection was 666 fluorescence (a.u), which resulted in detectable protease activity from 6.25 ng protein/mL, and continued to increase with higher protein concentrations (Figure 3Ci). The addition of a protease inhibitor cocktail successfully knocked out proteolytic activity, until 3,000 ng protein/mL.

As protease solutions were used every 2 h to stimulate MucilAir™ inserts for 8 h, the optimal storage conditions of the solution were determined. Four different concentrations of protease solution were tested at room temperature (RT) and at 4 °C for 8 h. The protease activity test indicates degradation of protease when the solutions are kept at RT rather than at 4 °C, as indicated by reduced proteolytic activity (Figure 3Cii). Thus, protease solutions were kept at 4 °C and equilibrated to RT before adding to cells.

### Epithelial barrier integrity

MucilAir™ inserts were apically exposed to a range of 0.00125–7,812 μg/mL protease for 8 h, re-stimulating every 2 h. A PBS control (0 μg/mL protease) was used as a negative control. Each donor was exposed to a selection of these concentrations in triplicate wells.

**FIGURE 4:**
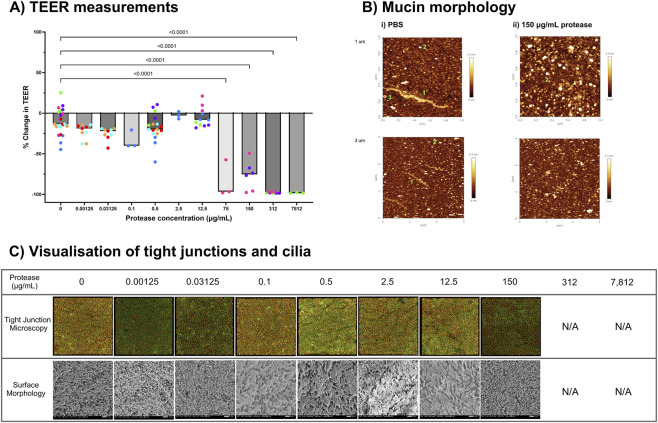
Epithelial barrier integrity. **(A)** The percentage change in TEER in each MucilAir™ insert from 0 h to 24 h of each exposure. A one-way ANOVA with Dunnett’s multiple comparisons compared the percentage change in TEER after each exposure to the PBS (0) control. N = 7 donors, with three replicate inserts measured for each exposure. Each donor is colour coded. **(B)** PBS-treated (I) and protease-treated (ii) epithelial cells were washed apically with PBS and the apical layer collected. Mucin molecules were then visualised with AFM at 1 μm and 3 µm. Label 1 = putative mucin molecule or a complex with DNA, label 2 = protein aggregates, label 3 = unidentified molecule. **(C)** Tight junction microscopy and surface morphology of MucilAir™ inserts exposed to 0–7,812 μg/mL protease. N/A = inserts degraded and so analysis could not be conducted.

TEER measurements were taken prior to exposure (0 h) and 24 h after the first dose. By normalising the 24 h TEER reading of each insert to its 0 h reading, the percentage change in TEER is presented (Figure 4A). Each percentage change in TEER from each exposure was then compared to the percentage change in the negative control. No significant differences in TEER above those occurring in the PBS control were noted after protease exposures of up to 12.5 μg/mL. Notably, TEER measurements then began to significantly decrease after exposures of 75–7,812 μg/mL protease (p < 0.0001).

To understand the impact of protease on the protective mucus layer, the morphology of mucin was first measured in a single apical wash from MucilAir™ inserts exposed to either PBS or 150 μg/mL protease, using Atomic Force microscopy (AFM). Figure 4B shows the presence of putative mucin molecules (either single or a supramolecular aggregate or a complex with DNA) in the PBS sample, but the mucin molecules were not present in the 150 μg/mL protease sample.

To assess tight junction degradation, 24 h after the first treatment dose, supernatants were removed, and the epithelial cells were fixed for tight junction staining. Immunofluorescence staining for Occludin and ZO-1 shows intact tight junctions for epithelial barriers exposed to up to 150 μg/mL of protease (Figure 4C). Hence, the protease solution does not appear to be affecting tight junction integrity at these concentrations. The MucilAir™ inserts completely detached at protease concentrations of 312 μg/mL and 7,812 μg/mL, thus the immunofluorescence staining could not be conducted.

After confocal microscopy of the tight junctions, a separate batch of inserts were used for cilia morphology visualisation by scanning electron microscopy (Figure 4C). Healthy cilia were observed after all exposures, apart from in concentrations above 312 μg/mL of protease, where the inserts were again too damaged for analysis.

### Cytokine production

**FIGURE 5:**
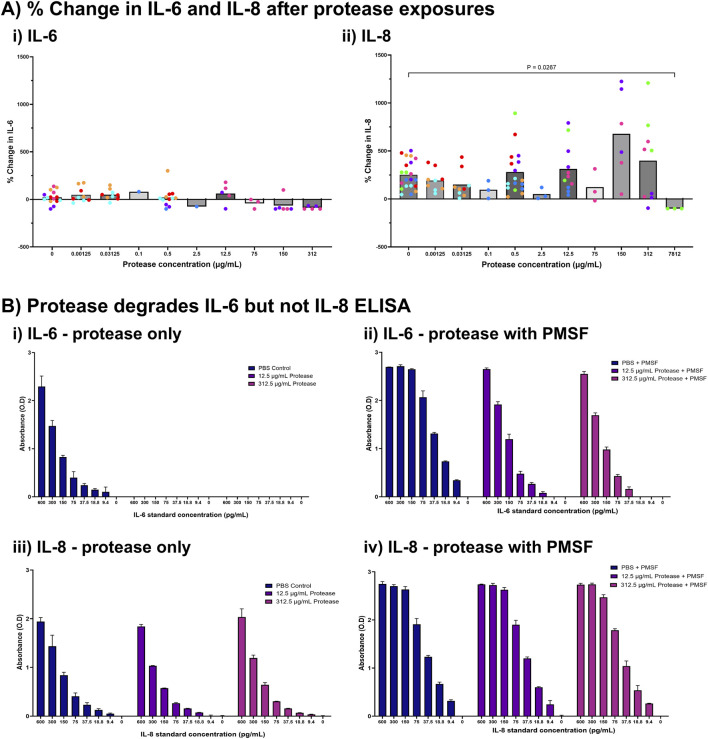
MucilAirTM cytokine production. **(A)** The percentage change in (I) IL-6 and (ii) IL-8 production in each MucilAir™ insert from 0 h to 24 h of each exposure. A Kruskal Wallis test with Dunnett’s multiple comparisons compared the percentage change in cytokine production after each exposure to the PBS (0) control. N = 7 donors, with three replicate inserts measured for each exposure. Each donor is colour-coded. **(B)** ELISA kit degradation was tested by performing (I) IL-6 ELISAs using IL-6 standards with protease and (ii) with protease + PMSF. (I) IL-8 ELISAs were also conducted using IL-8 standards with protease and (ii) with protease + PMSF. N = 3.

Epithelial cells produce cytokines in response to damage or other stimuli, including IL-6 and IL-8. As such, IL-6 and IL-8 cytokines secreted into the basal compartment of the MucilAir™ inserts were measured by ELISA after each protease exposure. As described, there was no significant change in expression of IL-6 over 24 h with PBS alone nor across any of the protease concentrations tested, as shown in Figure 5Ai.

Figure 5Aii shows the percentage change in basal IL-8 production after each exposure. The percentage change in IL-8 was higher for all treatments, except for the highest exposure of 7,812 μg/mL protease, which was significantly lower compared to the PBS control (p = 0.0267), likely due to rapid cell death. The increases in IL-8 production even in the PBS control-treated wells (mean = 252.6%, SD = 148.7) highlights potential background stimulation, which could be due to the action of the repeated dosing and the simple act of covering the cells in a liquid layer, reducing air exchange.

To examine if any residual protease activity in the supernatants was interfering with the ELISAs, either PBS, 12.5 μg/mL, or 312 μg/mL of protease was added to IL-6 and IL-8 standards. PMSF (0.2 mM) was then added to half of the standards for 1 h to block activity before conducting the ELISA. Figure 5Bi highlights 12.5 μg/mL and 312 μg/mL of active protease totally diminished the IL-6 detected in the standards. Figure 5Bii shows the restoration of the IL-6 when the PMSF was added. Interestingly, Figure 5Biii-iv shows that the active protease did not interfere with the IL-8 ELISA, and unsurprisingly, the PMSF did not alter the results. Thus, the IL-6 results generated are likely impacted by protease interference with the IL-6 ELISA. Future experiments would include incubation of supernatants with 0.2 mM of PMSF before adding to any cytokine assays to prevent interference.

### LDH production

**FIGURE 6:**
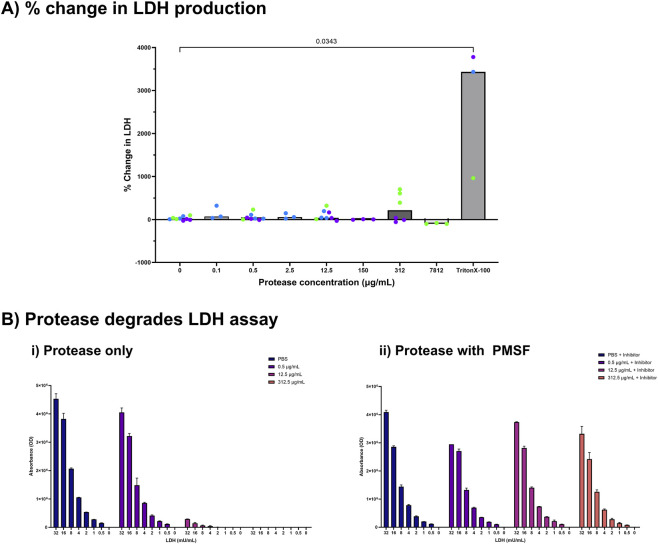
LDH production. **(A)** The percentage change in LDH production in each MucilAir™ insert from 0 h to 24 h of each exposure. A Kruskal Wallis test with Dunnett’s multiple comparisons compared the percentage change in cytokine production after each exposure to the PBS (0) control. N = 3 donors, with three replicate inserts measured for each exposure. Each donor is colour coded. **(B)** LDH standards were mixed with either PBS, 0.5, 12.5, or 312 μg/mL of protease and then incubated without (I) PMSF or with PMSF (ii) and luminescence was recorded. N = 3.

LDH production was measured to determine the cell viability of MucilAir™ inserts (N = 3) after protease exposures. The results show no significant increases in LDH release, apart from in the 10% triton X-100 positive control (p = 0.0343) (Figure 6A). These results were surprising, as some of the inserts visibly degraded at the highest protease concentrations. Thus, it was suspected that, as seen with the IL-6 assay, there may be some protease interference with the LDH assay.

As before, interference of protease was measured by adding either PBS, 0.5, 12.5, or 312 μg/mL of protease to LDH standards. PMSF (0.2 mM) was then added to half of the standards for 1 h before conducting the assay. Figure 6B demonstrates that LDH was not affected at 0.5 μg/mL of protease but was totally diminished with 12.5 and 312 μg/mL of protease. The LDH was detectable when PMSF was added to stop protease activity, showing interference when active protease is present. Again, future experiments would include incubation of supernatants with 0.2 mM of PMSF before adding to the assay to prevent interference.

### EV characterisation

**FIGURE 7:**
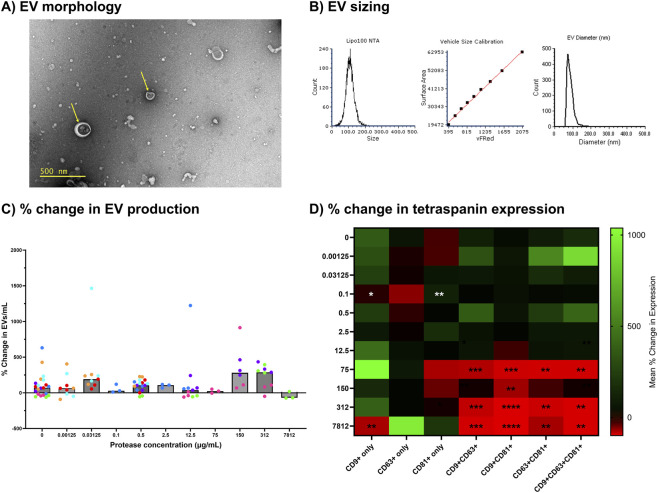
EV characterisation. **(A)** EVs derived from MucilAir™ inserts were isolated by SEC and imaged by TEM. **(B)** The imaging FC, ImageStream X MkII, employed a vesicle size standard confirmed by NTA to then be used for calibration with a fluorescent membrane stain. The calibration file could then be applied to MucilAir™ EV samples stained with the vFRed dye for sizing. **(C)** The percentage change in EV production in each MucilAir™ insert from 0 h to 24 h of each exposure. A Kruskal Wallis test with Dunn’s multiple comparisons compared the percentage change in EV production after each exposure to the PBS (0) control. N = 7 donors, with three replicate inserts measured for each exposure. Each donor is colour coded. **(D)** Calcein + EVs from each sample were stained for tetraspanin antibodies anti-CD9, -CD63, and -CD81. The mean percentage change in the number of EVs expressing each tetraspanin from 0 h to 24 h is presented. Different combinations of tetraspanin expressed on EVs are along the x-axis, and the exposures are along the y-axis. N = 7, with three replicate inserts measured for each exposure. A mixed-effects analysis test with Dunnett’s multiple comparisons was conducted: *p < 0.05, **p < 0.01, ***p < 0.001, ****p < 0.0001.

In agreement with MISEV guidelines (Welsh et al., 2024), the presence of EVs from MucilAir™ inserts were confirmed by TEM (Figure 7A). EVs were first isolated by size exclusion chromatography (SEC) and then imaged by TEM, confirming the presence of small EVs (<200 nm) in the cell supernatant, showing spherical, cup-shaped, membrane enclosed particles consistent with the morphology of EVs.

In addition, to confirm EV size, a representative basal supernatant sample was stained with the membrane dye vFRed™ (Figure 7B). Using a synthetic size standard (Lipo100), which has a size range of 80–140 nm as determined by NTA, the size of EVs stained with vFRed™ were calibrated using FCS Express transformations tool and inputting a surface area equation generated by the manufacturer’s layout. The MucilAir™ sample demonstrates EVs as small as 55 nm were present, with a mean size of 88 nm.

All MucilAir™ basal supernatants were stained with calcein-AM to quantify EVs, as well as CD9, CD63, and CD81 to measure their tetraspanin expression by flow cytometry. Figure 7C shows no significant differences in the percentage change in EVs/mL between the PBS control and protease exposures. Although, there is a trend of increased EV production at 150–312 μg/mL of protease, which then decreases at the 7,812 μg/mL protease exposure, likely due to rapid cell death. In terms of tetraspanins expression, there is a clear decrease in overall expression after 75–7,812 μg/mL of protease exposures (Figure 7D).

### Modelling the impact of protease exposures on TEER

**FIGURE 8:**
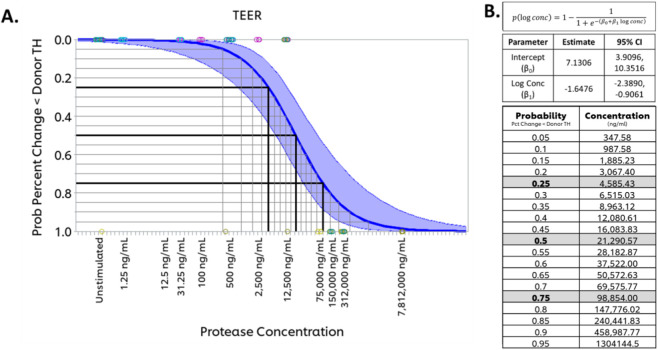
TEER probability modelling. **(A)** A logistic (binary response) curve to model the percentage change in TEER data. **(B)** Equations used to calculate the probability of a TEER change compared to unstimulated over 24 h at each protease concentration. N = 7.

Focusing on TEER measurements, as the data was most complete and less impacted by factors such as residual protease effects on the assay, the MucilAir™ response to protease treatment was modelled. The TEER data were used to calculate the probability of TEER decreasing when exposed to protease solutions. Figure 8A shows the use of a logistic (binary response) model to fit the TEER data. A threshold was determined from the unstimulated values, separately for each donor, by taking the P25–1.5 x IQR (a standard way of determining values that are below expected levels). The equations in Figure 8B were then used to determine the probability of TEER differing from unstimulated after different protease exposures, i.e., there is a 25% probability of protease resulting in a decreased TEER measurement at a concentration of 4,585.43 ng/mL.

## Discussion

There is a growing need for robust approaches for chemical risk assessments without the use of animals. ALI human lung models are a physiologically relevant system and are becoming a popular tool for inhalation experiments. Thus, in this study, use of a human ALI model of differentiated bronchial epithelium (MucilAir™) was assessed for investigating protease-induced effects. Our approach, based on attempts to extrapolate from inhalable airborne concentration to *in vitro* tissue concentrations to guide dosing concentrations, combined with repeated apical liquid dosing over 8 h, allowed us to explore, for the first time, concentration-dependent effects of protease exposure on epithelial barrier function, cytokine responses, and EV dynamics.

Enzymes introduced into detergent products in the 1960s/70 s initially led to high occupational exposures (up to the μg per m^3^ range), leading to high sensitisation rates (e.g., 40%–50%) in some worker populations and development of symptoms in a proportion of these, including rhinitis, conjunctivitis, and asthma (Sarlo and Kirchner, 2002; Schweigert et al., 2000). Introduction of exposure controls and occupational exposure limits (OELs) (e.g., the American Conference of Governmental and Industrial Hygienists (ACGIH) proposed a ceiling threshold limit value (TLV) of 60 ng/m^3^ for subtilisins in 1973, which was adopted in 1974) has minimised sensitisation and prevented symptoms (Sarlo, 2003; Basketter et al., 2021; Basketter et al., 2015). Additionally, a benchmarking approach underpins the risk assessment of enzyme containing consumer products, drawing on derived minimal effect levels (DMELs), with a DMEL of 15 ng per m^3^ typically proposed as a starting point for consumer product risk assessment (Basketter et al., 2012). The latest A.I.S.E. risk assessment guidance (AESI, 2025) also notes that actual air concentrations of enzymes during product use depend on formulation viscosity, enzyme loading, and user habits, all of which influence aerosolisation (including particle size) and particle dispersion. The document also provides details of some measured exposures and clinical outcomes among consumers using enzyme containing products illustrating the reduction in exposures and associated risks, as products developed over time.

Thus, using the MPPD model with refinements, this study utilised protease concentrations covering those associated with a low to high risk of allergic sensitisation in an occupational setting (8 h exposure to <1–10,000 ng protease per m^3^), which extrapolated to tracheobronchial tissue dose concentrations of <0.0002–2 ng/mL, to guide initial dosing.

It should be noted, however, that use of the MPPD model to guide protease exposure scenarios has its limitations and as such extrapolated values have a high level of associated uncertainty. The model relies on simplified and idealised airway geometries and deterministic airflow patterns, which do not fully capture the complex anatomical variability and dynamic conditions of the human lung, particularly in the alveolar regions where localised deposition can differ markedly (Asgharian et al., 2001). Furthermore, most inhalation models are designed on the *in vivo* lung environment and currently lack integration with key features of *in vitro* exposure platforms. For instance, for semi-submerged ALI cultures, models such as MPPD cannot describe the partitioning between media and cells, nor the dynamics of particle deposition in *vitro* scenarios (Haber et al., 2024). These limitations introduce considerable uncertainty when extrapolating MPPD outputs to *in vitro* exposure scenarios. As such, the use of hybrid modelling strategies, combining MPPD with computational fluid-particle dynamics (CFPD) and cell-specific *in vitro* dosimetry approaches, is increasingly necessary to ensure physiologically relevant dose estimations and more accurate risk assessments (Haber et al., 2024). In addition, although the MucilAir™ model used in this study reproduces key features of the airway epithelium, including differentiation, barrier integrity, and mucociliary function, it lacks aspects such as airflow, complex airway geometry, and immune-epithelial interactions.

Whilst noting the uncertainties associated with the extrapolated doses, we highlight no protease-related effects were observed at concentrations up to 500 ng/mL protease after apical liquid dosing in this MucilAir^TM^-bronchial model, with no significant changes to TEER, tight junctions, cilia morphology, cytokines, cell viability or EVs, when comparing to the PBS control. Biological variability between MucilAir™ donors and insert batches was accounted for using repeated-measures and mixed-effects analyses, with percentage changes from baseline normalising donor-specific responses. While some non-significant findings may reflect limited donor numbers or assay interference, consistent trends across donors and replicates support the reported exposure-response patterns. This shows that using this model and approach, protease exposures several orders of magnitude above those extrapolated from inhalable concentrations spanning low to high risk of sensitisation in an occupational setting (Basketter et al., 2012), did not cause any significant treatment-related effects. Hence, the findings provide further reassurance that current occupational exposure limits and the benchmarking approach used for consumer risk assessment are protective, as reflected in multiple studies, as these would lead to extrapolated tissues exposures (even when accounting for a high degree of uncertainty) far below those at which barrier disruption was measured. It is noted, however, that whilst the long-standing OEL of 60 ng/m^3^ for protease (subtilisin) set by ACGIH is for pure enzyme, industry practices aim for lower exposures than this to account for co-exposure to other detergent constituents (Basketter et al., 2010), and this was not explored in this study.

Furthermore, the lack of significant results at the low concentrations should be interpreted cautiously, as comparisons were made to the PBS control, in which we observed evidence of effects on the cells. These effects are attributable to repeated apical liquid application, whereby epithelial cells are effectively ‘drowning’ due to the unintended transient submersion of ALI cultures. The MucilAir™ inserts exhibited a significant increase in IL-8 production after 24 h of exposure to PBS. In addition, the epithelial barrier integrity was reduced, as indicated by a decrease in TEER measurement. EV production by the MucilAir™ inserts were also increased after 24 h of exposure to the PBS control, as well as the number of EVs expressing CD9. This highlights the limitations of adopting repeated apical liquid exposure, as it is compromising baseline epithelial homeostasis. Subsequently, statistical differences to the protease exposures may be reduced due to these background effects. This is consistent with other research using liquid exposures with bronchial cells, which also highlight PBS exposure impacting cells, including significant reprogramming of the transcriptome and biological pathway activity, leading to alternative regulation of cellular signalling pathways, increased secretion of pro-inflammatory cytokines and growth factors, and decreased epithelial barrier integrity (Mallek et al., 2024). The persistence of apical liquid between dosing intervals raises the possibility that repeated transient submersion may contribute to the observed baseline activation. Sustained surface coverage could alter airway surface liquid composition, disrupt mucociliary dynamics, or affect epithelial oxygenation, although these parameters were not directly measured in the present study. In this context, aerosolised delivery approaches may better preserve physiological ALI conditions by avoiding bulk liquid submersion and more closely mimicking inhalation exposure, and have been successfully used for evaluating test materials in ALI cultures by other researchers (de Ávila et al., 2025). However, direct comparative studies would be required to determine whether such approaches mitigate baseline activation.

When moving beyond the dosing concentrations extrapolated to cover the indicated inhalable airborne concentrations, to understand protease levels that do induce a significant effect in the cell model and with the dosing approach described, we showed that from 75 μg protease/mL, there was a significant reduction in TEER measurement, which continued to decrease at higher concentrations. Further statistical and modelling analysis highlight that fitting a binary response model to the TEER data suggests a 75% probability of altered TEER measurements at 98.9 μg/mL, and a 25% probability at 4.6 μg/mL)

Direct studies involving proteases on ALI cultures are limited. None could be found using MucilAir™ or other 3D epithelial models. A similar study using human CFBE41o-bronchial epithelial cells found a significant decrease in TEER after 10 h of serine protease exposure, derived from the Gram-negative bacteria *Stenotrophomonas maltophilia,* which had a protease activity of 5 × 10^3^ RFU/min (Molloy et al., 2020). Other work involving Der p 1, a cysteine protease, clearly indicates tight junction cleavage, though this used human nasal epithelial cells from patients with allergic rhinitis (Ogi et al., 2021), or Madin-Darby canine kidney (MDCK) and 16HBE14o^–^human bronchial epithelial cell lines (Wan et al., 1999) and whole HDM fecal pellet exposure, and different effects across defined protease exposures could not be compared.

In addition to changes in TEER, apical AFM imaging revealed that mucin, which is critical for forming a protective barrier, was visibly degraded at 150 μg/mL protease, indicating that high concentrations of proteases can erode the mucosal layer before deeper tissue effects manifest. However, immunofluorescent staining indicated that tight junction proteins remained intact up to 150 μg/mL protease, but at higher doses, the inserts degraded and could not be analysed for tight junction integrity.

This suggests that the presence of mucus in this 3D model may prevent tight junction disruption at lower exposures, and that mucin degradation may precede tight junction disruption, a sequential injury model not always captured in simpler epithelial models. The results are supported by previous work, where mucin degradation noted in cystic fibrosis patients infected with *P. aeruginosa* was linked to serine proteases released by the bacteria, which degraded MUC5AC and MUC5B (Henke et al., 2011). This work also highlights the importance of further understanding (and building in) the role of mucus in inhalation exposure models. Mucus clearance of materials a critical parameter but as this works illustrates can be impacted by inhaled materials with potential interactions such as degradation in the case of enzymes like proteases.

EVs were also measured in this study, as they have been recently identified as key modulators in airway immune responses (Browne et al., 2025; Ambrożej et al., 2022b; Holtzman and Lee, 2020). EVs function as signalling molecules, containing information such as miRNAs, cytokines chemokines. Because of their well-documented involvement in respiratory diseases, EVs show promise both as possible diagnostic biomarkers and as therapeutic agents. Here, we show all MucilAir™ donors produced EVs as detected by flow cytometry staining. There were trends of increased EV production after 150 μg/mL and 312 μg/mL of protease, while CD9, CD63, and CD81 tetraspanin expression decreased at these high concentrations, likely due to protease degradation of the surface markers. The loss of tetraspanin expression after high protease exposures is likely to affect the biological functions of the EVs, as tetraspanins are required for roles such as regulating endosomal network dynamics and EV biogenesis/cargo selection (Toribio and Yáñez-Mó, 2022).

MucilAir™ epithelia have previously been shown to secrete both IL-6 and IL-8 (Welch et al., 2021). However, contrary to expectations, basal IL-6 and IL-8 secretion did not significantly increase following high protease exposures. For IL-6, in addition to donor variability at baseline, the lack of a measurable effect was likely due to proteolytic degradation of the ELISA detection reagents. Any residual protease in the basal compartment after exposures was found to degrade the ELISA, as confirmed by spiking experiments showing undetectable IL-6 unless protease inhibitors (PMSF) were added. The same was noted for the LDH assay, where any residual protease at concentrations above 12.5 μg/mL of protease degraded the assay. Thus, apparent “viability” must be interpreted cautiously, as assay interference likely masked true cell damage at higher protease concentrations. This highlights an important caveat; assays measuring soluble biomarkers in protease-exposed supernatants may be subject to analytical interference, and thus these IL-6 and LDH results are not true biological findings. Future studies will include protease inhibition, such as PMSF, in supernatants to preserve assay integrity. The IL-8 ELISA appeared unaffected by residual protease interference and showed that MucilAir™ inserts exposed to the highest concentration of 7,812 μg/mL of protease produced significantly less IL-8 than the PBS control, likely due to rapid cell death.

Despite observing effects on the epithelial barrier at 75 μg/mL of protease and above, the stated concentrations could be over-estimated, as protease solutions can undergo autolysis, breaking themselves down over time (Jiang et al., 2021; Stoner et al., 2004). This self-cleavage may reduce the effective concentration and activity of the enzyme during the exposure, making it difficult to interpret exposure-response relationships. This is amplified by the presence of endogenous proteases and inhibitors in the bronchial epithelium, which have the ability to cleave serine proteases to further decrease their activity (McKelvey et al., 2021; Soh et al., 2023). Future studies could implement a control to measure any decline in protease activity over the 8 h exposure window, to factor into calculating representative dose-responses.

In summary, our study provides an important step toward quantifying protease-induced airway epithelial damage in a human-relevant, *in vitro* system. Importantly, we identify issues with apical liquid exposures, with effects observed in the negative control and hypothesise moving to a nebulised delivery system may overcome this. We also further reinforce the need for improved human exposure datasets and available exposure models for proteins, including enzymes, which also need to be developed with outputs designed to guide *in vitro* dosing. We highlight challenges that can be encountered when working with enzymes. Despite the challenges and limitations, we showed that with the approach used, modelling of TEER data suggests a 75% probability of altered TEER measurements at exposures of 98.9 μg protease protein/mL, and a 25% probability at 4.6 μg/mL. While cytokine and viability assays were confounded by protease interference, EV dynamics offered additional insight into cellular stress responses at high protease concentrations. We conclude that protease exposures can elicit a sequential response, with intermediate concentrations inducing acute epithelial stress, such as mucin degradation and altered EV release, while higher concentrations cause overt cytotoxicity, including barrier collapse. These effects however occurred at concentrations far exceeding those modelled to reflect occupational exposures, providing further reassurance that current risk management practices are protective with exposures kept below those that led to barrier disruption *in vitro*, in this study. Overall, this study offers many insights for the development of *in vitro* approaches to support risk assessment of inhalation of proteases and other proteins and guide further work.

## Data Availability

The raw data supporting the conclusions of this article will be made available by the authors, without undue reservation.

## References

[B1] Adisesh A. Murphy E. Barber C. M. Ayres J. G. (2011). Occupational asthma and rhinitis due to detergent enzymes in healthcare. Occup. Med. 61 (5), 364–369. 10.1093/occmed/kqr107 21831827

[B2] Aesi A. C. I. (2025). Guidance for the Risk Assessment of enzyme-containing Consumer Products. Available online at: https://www.cleaninginstitute.org/sites/default/files/documents/AISE-ACI-Guidance-Risk-Assessement-Enzyme-Containing-Consumer-Products.pdf (Accessed June 26, 2025).

[B3] Akdis C. A. (2021). Does the epithelial barrier hypothesis explain the increase in allergy, autoimmunity and other chronic conditions? Nat. Rev. Immunol. 21 (11), 739–751. 10.1038/s41577-021-00538-7 33846604

[B4] Allouche Y. Marchetti S. Bengalli R. Motta G. Pagliarulo L. Cazier F. (2025). Comparison of submerged and air liquid interface exposure: limitations and differences in the toxicological effects evaluated in bronchial epithelial cells. Environ. Res. 279, 121856. 10.1016/j.envres.2025.121856 40379004

[B5] Ambrożej D. Stelmaszczyk-Emmel A. Czystowska-Kuźmicz M. Feleszko W. (2022a). “Liquid biopsy” - extracellular vesicles as potential novel player towards precision medicine in asthma. Front. Immunol. 13, 1025348. 10.3389/fimmu.2022.1025348 36466836 PMC9714548

[B6] Ambrożej D. Stelmaszczyk-Emmel A. Czystowska-Kuźmicz M. Feleszko W. (2022b). “Liquid biopsy” - extracellular vesicles as potential novel players towards precision medicine in asthma. Front. Immunol., 13–2022. 10.3389/fimmu.2022.1025348 PMC9714548 36466836

[B7] Asgharian B. Hofmann W. Bergmann R. (2001). Particle deposition in a multiple-path model of the human lung. Aerosol Sci. Technol. 34 (4), 332–339. 10.1080/02786820119122

[B8] Ax E. Jevnikar Z. Cvjetkovic A. Malmhäll C. Olsson H. Rådinger M. (2020). T2 and T17 cytokines alter the cargo and function of airway epithelium-derived extracellular vesicles. Respir. Res. 21 (1), 155. 10.1186/s12931-020-01402-3 32560723 PMC7304225

[B9] Balharry D. Sexton K. BéruBé K. A. (2008). An *in vitro* approach to assess the toxicity of inhaled tobacco smoke components: nicotine, cadmium, formaldehyde and urethane. Toxicology 244 (1), 66–76. 10.1016/j.tox.2007.11.001 18082304

[B10] Basketter D. A. Broekhuizen C. Fieldsend M. Kirkwood S. Mascarenhas R. Maurer K. (2010). Defining occupational and consumer exposure limits for enzyme protein respiratory allergens under REACH. Toxicology 268 (3), 165–170. 10.1016/j.tox.2009.12.014 20026217

[B11] Basketter D. Berg N. Broekhuizen C. Fieldsend M. Kirkwood S. Kluin C. (2012). Enzymes in cleaning products: an overview of toxicological properties and risk assessment/management. Regul. Toxicol. Pharmacol. 64 (1), 117–123. 10.1016/j.yrtph.2012.06.016 22743221

[B12] Basketter D. A. Kruszewski F. H. Mathieu S. Kirchner D. B. Panepinto A. Fieldsend M. (2015). Managing the risk of occupational allergy in the enzyme detergent industry. J. Occup. Environ. Hyg. 12 (7), 431–437. 10.1080/15459624.2015.1011741 25692928 PMC4806342

[B13] Basketter D. Moreno N. Simonsen M. (2021). Occupational exposure limits for enzymes: practical considerations. Int J Pul and Res Sci. 5 (1), 555651. 10.19080/IJOPRS.2021.05.555651

[B14] Browne W. Hopkins G. Cochrane S. James V. Onion D. Fairclough L. C. (2025). The role of epithelial-derived extracellular vesicles in allergic sensitisation: a systematic review. Int. J. Mol. Sci. 26 (12), 5791. 10.3390/ijms26125791 40565252 PMC12193434

[B15] Cochrane S. Rajagopal R. Sheffield D. Stewart F. Hathaway L. Barnes N. M. (2024). Impact of a varied set of stimuli on a suite of immunological parameters within peripheral blood mononuclear cells: toward a non-animal approach for assessing immune modulation by materials intended for human use. Front. Toxicol. 6–1335110. 10.3389/ftox.2024.1335110 38737195 PMC11082367

[B16] David B. J. C. Dill D. Forsythe S. Holtkoetter O. (2024). Is allergy to industrial enzymes used in household detergent products stable in the general population? Int J Pul and Res Sci 7 (3), 555714. 10.19080/IJOPRS.2024.07.555715

[B17] de Ávila R. I. Müller I. Barlow H. Middleton A. M. Theiventhran M. Basili D. (2025). Evaluation of a non-animal toolbox informed by adverse outcome pathways for human inhalation safety. Front. Toxicol. 7–1426132. 10.3389/ftox.2025.1426132 40061084 PMC11885506

[B18] Dent M. Amaral R. T. Da Silva P. A. Ansell J. Boisleve F. Hatao M. (2018). Principles underpinning the use of new methodologies in the risk assessment of cosmetic ingredients. Comput. Toxicol. 7, 20–26. 10.1016/j.comtox.2018.06.001

[B19] Dent M. P. Vaillancourt E. Thomas R. S. Carmichael P. L. Ouedraogo G. Kojima H. (2021). Paving the way for application of next generation risk assessment to safety decision-making for cosmetic ingredients. Regul. Toxicol. Pharmacol. 125, 105026. 10.1016/j.yrtph.2021.105026 34389358 PMC8547713

[B20] Flindt M. L. H. (1969). Pulmonary disease due to inhalation of derivatives of bacillus subtilis containing proteolytic enzyme. Lancet 293 (7607), 1177–1181. 10.1016/s0140-6736(69)92165-5 4181838

[B21] Haber L. T. Bradley M. A. Buerger A. N. Behrsing H. Burla S. Clapp P. W. (2024). New approach methodologies (NAMs) for the *in vitro* assessment of cleaning products for respiratory irritation: workshop report. Front. Toxicol., 6–1431790. 10.3389/ftox.2024.1431790 39439531 PMC11493779

[B22] Henke M. O. John G. Rheineck C. Chillappagari S. Naehrlich L. Rubin B. K. (2011). Serine proteases degrade airway mucins in cystic fibrosis. Infect. Immun. 79 (8), 3438–3444. 10.1128/IAI.01252-10 21646446 PMC3147599

[B23] Holtzman J. Lee H. (2020). Emerging role of extracellular vesicles in the respiratory system. Exp. and Mol. Med. 52 (6), 887–895. 10.1038/s12276-020-0450-9 32541816 PMC7338515

[B24] Jiang L. Yuan C. Huang M. (2021). A general strategy to inhibit serine protease by targeting its autolysis loop. Faseb J. 35 (2), e21259. 10.1096/fj.202002139RR 33417271

[B25] Lujan H. Criscitiello M. F. Hering A. S. Sayes C. M. (2019). Refining *in vitro* toxicity models: comparing baseline characteristics of lung cell types. Toxicol. Sci. 168 (2), 302–314. 10.1093/toxsci/kfz001 30657991

[B26] Mallek N. M. Martin E. M. Dailey L. A. McCullough S. D. (2024). Liquid application dosing alters the physiology of air-liquid interface (ALI) primary human bronchial epithelial cell/lung fibroblast co-cultures and *in vitro* testing relevant endpoints. Front. Toxicol., 5–, 1264331. 10.3389/ftox.2023.1264331 38464699 PMC10922929

[B27] McKelvey M. C. Brown R. Ryan S. Mall M. A. Weldon S. Taggart C. C. (2021). Proteases, mucus, and mucosal immunity in chronic lung disease. Int. J. Mol. Sci. 22 (9), 5018. 10.3390/ijms22095018 34065111 PMC8125985

[B28] Molloy K. Cagney G. Dillon E. T. Wynne K. Greene C. M. McElvaney N. G. (2020). Impaired airway epithelial barrier integrity in response to Stenotrophomonas maltophilia proteases, novel insights using cystic fibrosis bronchial epithelial cell secretomics. Front. Immunol. 11, 198. 10.3389/fimmu.2020.00198 32161586 PMC7053507

[B29] Naidenko O. V. Andrews D. Q. Temkin A. M. Stoiber T. Uche U. I. Evans S. (2021). Investigating molecular mechanisms of immunotoxicity and the utility of ToxCast for immunotoxicity screening of chemicals added to food. Int. J. Environ. Res. Public Health 18 (7), 3332. 10.3390/ijerph18073332 33804855 PMC8036665

[B30] Noureddine N. Chalubinski M. Wawrzyniak P. (2022). The role of defective epithelial barriers in allergic lung disease and asthma development. J. Asthma Allergy 15, 487–504. 10.2147/JAA.S324080 35463205 PMC9030405

[B31] Ogi K. Ramezanpour M. Liu S. Ferdoush Tuli J. Bennett C. Suzuki M. (2021). Der p 1 Disrupts the Epithelial Barrier and Induces IL-6 Production in Patients With House Dust Mite Allergic Rhinitis. Front. Allergy 2, 2–2021. 10.3389/falgy.2021.692049 35387029 PMC8974687

[B32] Oh J.-H. Kim S.-H. Kwon O.-K. Kim J.-H. Oh S.-R. Han S.-B. (2022). Purpurin suppresses atopic dermatitis *via* TNF-α/IFN-γ-induced inflammation in HaCaT cells. Int. J. Immunopathol. Pharmacol. 36, 03946320221111135. 10.1177/03946320221111135 35794850 PMC9274433

[B33] Sarlo K. (2003). Control of occupational asthma and allergy in the detergent industry. Ann. Allergy Asthma Immunol. 90 (5 Suppl. 2), 32–34. 10.1016/s1081-1206(10)61646-8 12772949

[B34] Sarlo K. Kirchner D. B. (2002). Occupational asthma and allergy in the detergent industry: new developments. Curr. Opin. Allergy Clin. Immunol. 2 (2), 97–101. 10.1097/00130832-200204000-00003 11964756

[B35] Schweigert M. K. Mackenzie D. P. Sarlo K. (2000). Occupational asthma and allergy associated with the use of enzymes in the detergent industry--a review of the epidemiology, toxicology and methods of prevention. Clin. Exp. Allergy 30 (11), 1511–1518. 10.1046/j.1365-2222.2000.00893.x 11069558

[B36] Soh W. T. Zhang J. Hollenberg M. D. Vliagoftis H. Rothenberg M. E. Sokol C. L. (2023). Protease allergens as initiators-regulators of allergic inflammation. Allergy 78 (5), 1148–1168. 10.1111/all.15678 36794967 PMC10159943

[B37] Song P. Zhang X. Wang S. Xu W. Wang F. Fu R. (2023). Microbial proteases and their applications. Front. Microbiol., 14–1236368. 10.3389/fmicb.2023.1236368 37779686 PMC10537240

[B38] Stoner M. R. Dale D. A. Gualfetti P. J. Becker T. Manning M. C. Carpenter J. F. (2004). Protease autolysis in heavy-duty liquid detergent formulations: effects of thermodynamic stabilizers and protease inhibitors. Enzyme Microb. Technol. 34 (2), 114–125. 10.1016/j.enzmictec.2003.09.008

[B40] Toribio V. Yáñez-Mó M. (2022). Tetraspanins interweave EV secretion, endosomal network dynamics and cellular metabolism. Eur. J. Cell. Biol. 101 (3), 151229. 10.1016/j.ejcb.2022.151229 35500468

[B41] Tucis D. Hopkins G. Browne W. James V. Onion D. Fairclough L. C. (2024). The role of extracellular vesicles in allergic sensitization: a systematic review. Int. J. Mol. Sci. 25 (8), 4492. 10.3390/ijms25084492 38674077 PMC11049870

[B42] van Ree R. Hummelshøj L. Plantinga M. Poulsen L. K. Swindle E. (2014). Allergic sensitization: host-immune factors. Clin. Translational Allergy 4 (1), 12. 10.1186/2045-7022-4-12 24735802 PMC3989850

[B43] Wan H. Winton H. L. Soeller C. Tovey E. R. Gruenert D. C. Thompson P. J. (1999). Der p 1 facilitates transepithelial allergen delivery by disruption of tight junctions. J. Clin. Invest. 104 (1), 123–133. 10.1172/JCI5844 10393706 PMC408401

[B44] Wang X. Li N. Ma M. Han Y. Rao K. (2022). Immunotoxicity *in vitro* assays for environmental pollutants under paradigm shift in toxicity tests. Int. J. Environ. Res. Public Health 20 (1), 273. 10.3390/ijerph20010273 36612599 PMC9819277

[B45] Welch J. Wallace J. Lansley A. B. Roper C. (2021). Evaluation of the toxicity of sodium dodecyl sulphate (SDS) in the MucilAir™ human airway model *in vitro* . Regul. Toxicol. Pharmacol. 125, 105022. 10.1016/j.yrtph.2021.105022 34333067

[B46] Welsh J. A. Goberdhan D. C. I. O'Driscoll L. Buzas E. I. Blenkiron C. Bussolati B. (2024). Minimal information for studies of extracellular vesicles (MISEV2023): from basic to advanced approaches. J. Extracell. Vesicles 13 (2), e12404. 10.1002/jev2.12404 38326288 PMC10850029

