# Supplementary material for: Impact of liquid dosing and associated cell ‘drowning’ in a 3D human bronchial epithelial model used for protease exposure studies
Source: Front Toxicol. 2026 May 27;8:1781231. doi: 10.3389/ftox.2026.1781231 (PMC13265153; doi:10.3389/ftox.2026.1781231)
Supplement: Supplementary file 1 [file Supplementaryfile1.docx]

# **Choice of Initial Dose Range for Study**

**Aim:** To estimate tracheobronchial tissue exposures based on 8-hour occupational exposures to inhalable airborne protein concentrations of 1, 10, 1000 and 10,000 ng per m^3^**.**

**Method:** Using the MPPD model (v3.04) with refinement of Outputs for the Tracheobronchial (TB) region using a TB/Alveolar Clearance model.

- MPPD v3.04 was used with parameters as indicated in the screenshots below, varying the aerosol concentration to reflect those indicated in the aim above and to cover both 1 and 3 µM particle diameters. Deposition only using the Yeh-Schum human airway geometry model ( 3300 mL for Functional Residual Capacity (FRC) and 50 mL for Upper Respiratory Tract (URT) volume, the standard parameters used in occupational health and aerosol dosimetry studies for an average-sized adult male (ICRP 1994)) with lung geometry: Human symmetric. The influence of different tidal volumes was explored by simulating both 625mL and 1250mL volumes to reflect a sedentary adult and one undertaking light activity. Different breathing models (nasal only, oral only, oral-nasal augmented and oral-nasal mouth breather) were also simulated.


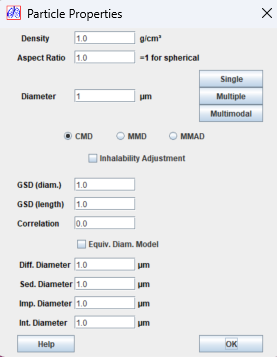


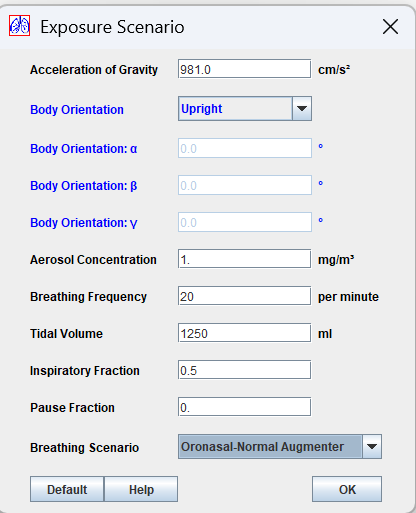


- This modelling with these parameters was considered to be a conservative approach based on a single, inhalable, particle size rather than distribution (hence the selection of CMD versus MMAD, though it is recognised that the MPPD model converts user-inputted count-based size metrics (like CMD) to mass-based metrics (like MMAD) internally when necessary to calculate particle deposition, provided the particle density and geometric standard deviation (GSD) are specified ), combined with default settings, such as low vapour pressure (typically defined in this context as having a vapor pressure less than 0.1 mmHg), as the model is not designed for protein exposures. Other particle characteristics such as hygroscopicity of particles were not explored, noting that variation of this property can lead to significant shifts in deposition patterns (Man et al 2022), thus it is recognised that any modelling outputs have associated uncertainties.
- The outputs from this modelling were then refined for the tracheobronchial region using a tracheobronchial/alveolar clearance model developed as described in de Avila *et al* 2025.
- *A priori* notes: The choice of 1 µM and 3 µM deposition values is valid for scenarios where both upper and lower airway deposition are valid concerns within the micron sized range. Submicron (0.01-0.1 µM) pulmonary deposition exceeds tracheobronchial (TB). In the nanometre range deposition in the upper airway/head once again exceeds pulmonary deposition at low values and then TB deposition dominates at 5 nm to 30 nm (see Figure 1). For the purposes of this evaluation 1 µM and 3 µM, 10 nM and 100 nM particle sizes were initially considered.


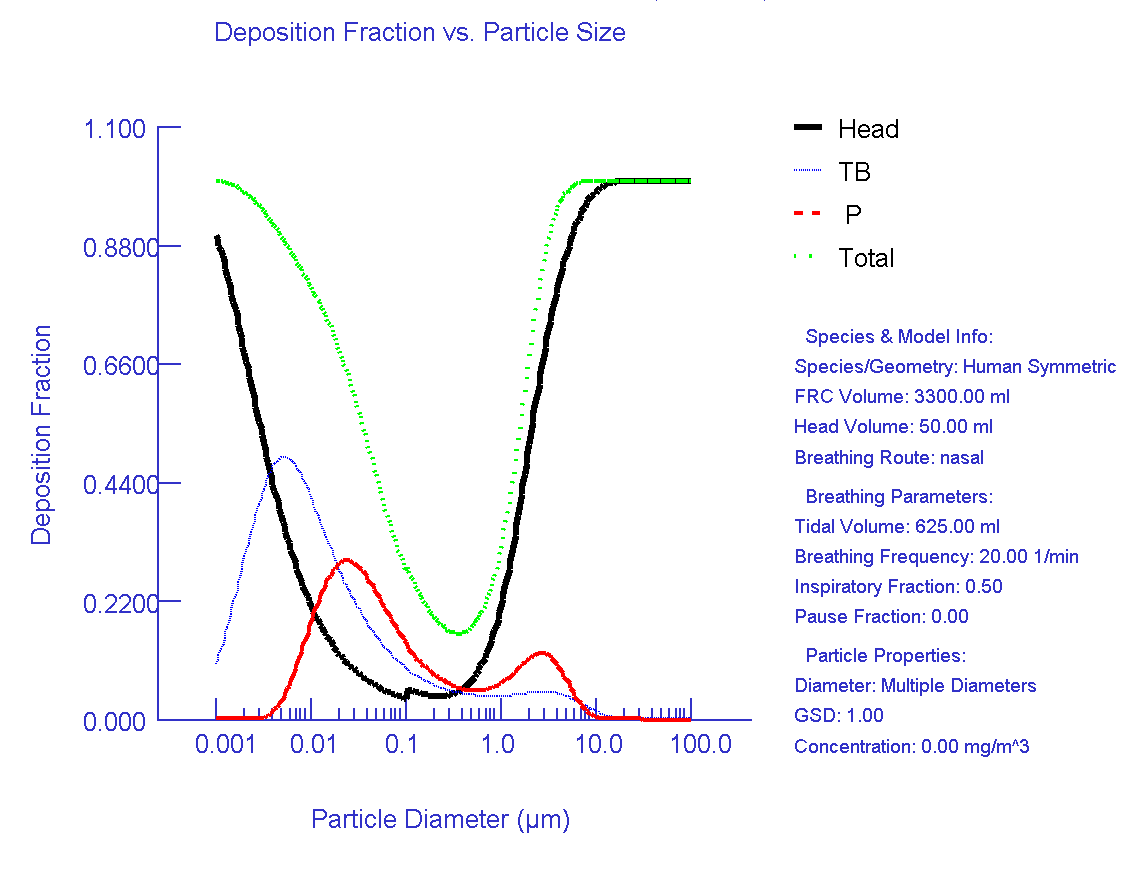

***Figure 1.*** *Deposition versus particle size in different regions of the respiratory tract.*

**Results**

The results (for an example graphic output see Figure 2) are shown for calculations based on daily exposure over the course of 2 weeks. Note: The nasal cavity is not included in the code, so the clearance calculations were only for the TB airway.


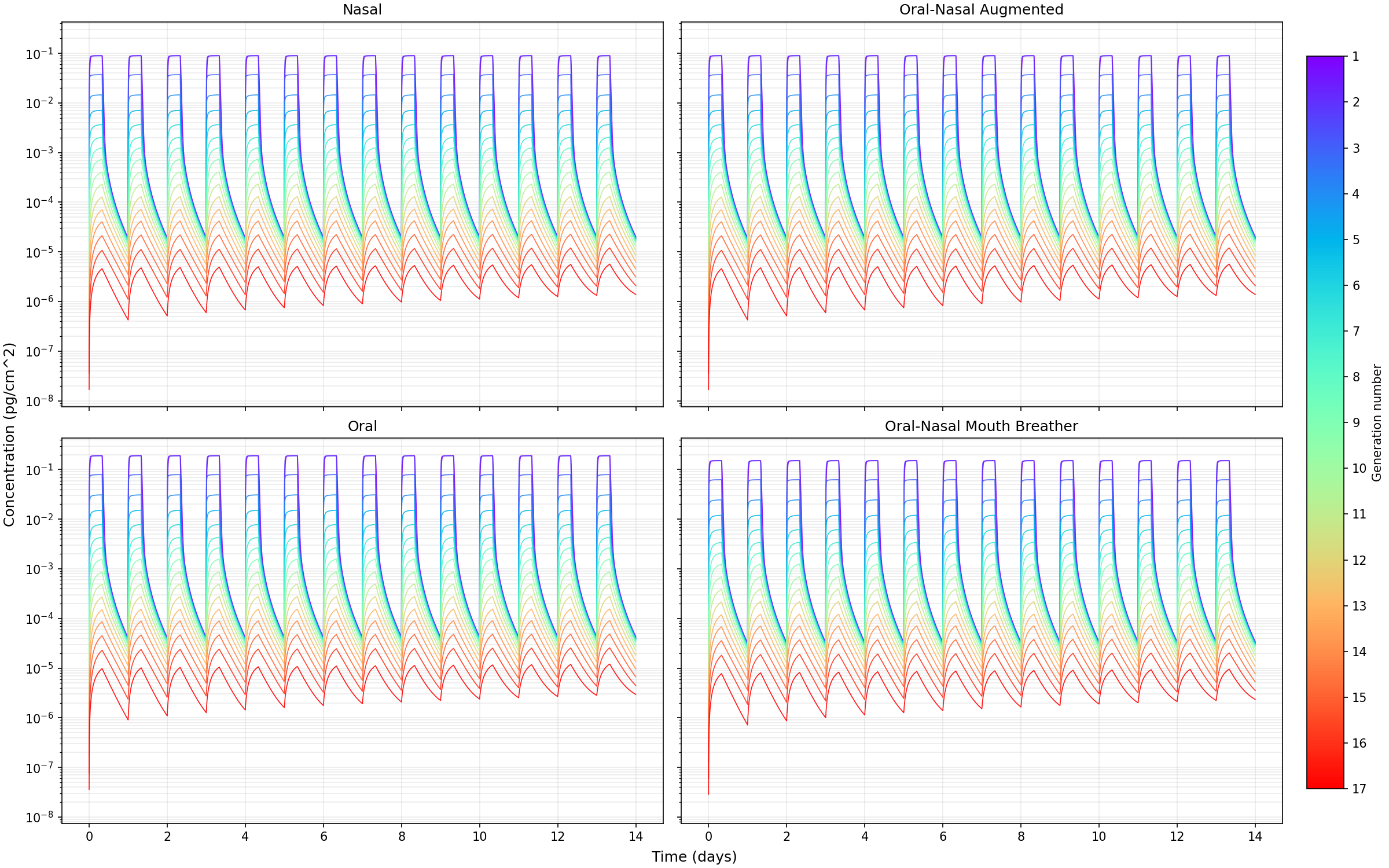


*Figure 2: Example plot of upper airway concentration for a 3 µM particle size, tidal volume of 625 ml, 8hr exposure, 20 breaths per minute for 10ng/m^3^ concentration. Trends show steady accumulation over the course of 2 weeks of daily 8-hour exposure.*

For each of the combinations of parameters a simulation was performed. In general, during exposure, the trachea is subjected to the highest local dose as all deposited material which is subsequently cleared must traverse the uppermost airways to be cleared.

This model’s main limitation is that it does not account for particle sizing or chemical effects. All clearance is assumed to be at a constant rate no matter the nature of the deposited particle.

In Table 2 are the results for 1 and 3µM particle sizes.

| **Particle Size (µm)** | **Tidal Breathing Volume (mL)** | **Breathing Scenario** | **Airborne Concentration** | **Max Surface Concentration (pg/cm^2^)** | **Corresponding Dosing Concentration (pg/ml)** |
| --- | --- | --- | --- | --- | --- |
| 1 | 625 | Nasal | 1 | 0.0139171 | 0.091852859 |
| 1 | 625 | Nasal | 10 | 0.139170998 | 0.918528586 |
| 1 | 625 | Nasal | 1000 | 13.91709979 | 91.85285863 |
| 1 | 625 | Nasal | 10000 | 139.1709979 | 918.5285863 |
| 1 | 625 | Oral | 1 | 0.015729368 | 0.103813828 |
| 1 | 625 | Oral | 10 | 0.157293678 | 1.038138276 |
| 1 | 625 | Oral | 1000 | 15.72936782 | 103.8138276 |
| 1 | 625 | Oral | 10000 | 157.2936782 | 1038.138276 |
| 1 | 625 | Oral-Nasal Augmented | 1 | 0.0139171 | 0.091852859 |
| 1 | 625 | Oral-Nasal Augmented | 10 | 0.139170998 | 0.918528586 |
| 1 | 625 | Oral-Nasal Augmented | 1000 | 13.91709979 | 91.85285863 |
| 1 | 625 | Oral-Nasal Augmented | 10000 | 139.1709979 | 918.5285863 |
| 1 | 625 | Oral-Nasal Mouth Breather | 1 | 0.015093279 | 0.099615643 |
| 1 | 625 | Oral-Nasal Mouth Breather | 10 | 0.150932792 | 0.996156427 |
| 1 | 625 | Oral-Nasal Mouth Breather | 1000 | 15.09327919 | 99.61564268 |
| 1 | 625 | Oral-Nasal Mouth Breather | 10000 | 150.9327919 | 996.1564268 |
| 1 | 1250 | Nasal | 1 | 0.0278342 | 0.183705717 |
| 1 | 1250 | Nasal | 10 | 0.278341996 | 1.837057173 |
| 1 | 1250 | Nasal | 1000 | 27.83419959 | 183.7057173 |
| 1 | 1250 | Nasal | 10000 | 278.3419959 | 1837.057173 |
| 1 | 1250 | Oral | 1 | 0.031458736 | 0.207627655 |
| 1 | 1250 | Oral | 10 | 0.314587356 | 2.076276552 |
| 1 | 1250 | Oral | 1000 | 31.45873564 | 207.6276552 |
| 1 | 1250 | Oral | 10000 | 314.5873564 | 2076.276552 |
| 1 | 1250 | Oral-Nasal Augmented | 1 | 0.0278342 | 0.183705717 |
| 1 | 1250 | Oral-Nasal Augmented | 10 | 0.278341996 | 1.837057173 |
| 1 | 1250 | Oral-Nasal Augmented | 1000 | 27.83419959 | 183.7057173 |
| 1 | 1250 | Oral-Nasal Augmented | 10000 | 278.3419959 | 1837.057173 |
| 1 | 1250 | Oral-Nasal Mouth Breather | 1 | 0.030186558 | 0.199231285 |
| 1 | 1250 | Oral-Nasal Mouth Breather | 10 | 0.301865584 | 1.992312854 |
| 1 | 1250 | Oral-Nasal Mouth Breather | 1000 | 30.18655839 | 199.2312854 |
| 1 | 1250 | Oral-Nasal Mouth Breather | 10000 | 301.8655839 | 1992.312854 |
| 3 | 625 | Nasal | 1 | 0.008973696 | 0.059226393 |
| 3 | 625 | Nasal | 10 | 0.089736959 | 0.59226393 |
| 3 | 625 | Nasal | 1000 | 8.973695906 | 59.22639298 |
| 3 | 625 | Nasal | 10000 | 89.73695906 | 592.2639298 |
| 3 | 625 | Oral | 1 | 0.019113653 | 0.126150113 |
| 3 | 625 | Oral | 10 | 0.191136535 | 1.261501128 |
| 3 | 625 | Oral | 1000 | 19.11365345 | 126.1501128 |
| 3 | 625 | Oral | 10000 | 191.1365345 | 1261.501128 |
| 3 | 625 | Oral-Nasal Augmented | 1 | 0.008973696 | 0.059226393 |
| 3 | 625 | Oral-Nasal Augmented | 10 | 0.089736959 | 0.59226393 |
| 3 | 625 | Oral-Nasal Augmented | 1000 | 8.973695906 | 59.22639298 |
| 3 | 625 | Oral-Nasal Augmented | 10000 | 89.73695906 | 592.2639298 |
| 3 | 625 | Oral-Nasal Mouth Breather | 1 | 0.015142876 | 0.09994298 |
| 3 | 625 | Oral-Nasal Mouth Breather | 10 | 0.151428758 | 0.999429802 |
| 3 | 625 | Oral-Nasal Mouth Breather | 1000 | 15.14287579 | 99.94298022 |
| 3 | 625 | Oral-Nasal Mouth Breather | 10000 | 151.4287579 | 999.4298022 |
| 3 | 1250 | Nasal | 1 | 0.017444338 | 0.115132632 |
| 3 | 1250 | Nasal | 10 | 0.174443382 | 1.151326323 |
| 3 | 1250 | Nasal | 1000 | 17.44433823 | 115.1326323 |
| 3 | 1250 | Nasal | 10000 | 174.4433823 | 1151.326323 |
| 3 | 1250 | Oral | 1 | 0.056801845 | 0.37489218 |
| 3 | 1250 | Oral | 10 | 0.568018455 | 3.748921803 |
| 3 | 1250 | Oral | 1000 | 56.80184549 | 374.8921803 |
| 3 | 1250 | Oral | 10000 | 568.0184549 | 3748.921803 |
| 3 | 1250 | Oral-Nasal Augmented | 1 | 0.017444338 | 0.115132632 |
| 3 | 1250 | Oral-Nasal Augmented | 10 | 0.174443382 | 1.151326323 |
| 3 | 1250 | Oral-Nasal Augmented | 1000 | 17.44433823 | 115.1326323 |
| 3 | 1250 | Oral-Nasal Augmented | 10000 | 174.4433823 | 1151.326323 |
| 3 | 1250 | Oral-Nasal Mouth Breather | 1 | 0.046296809 | 0.305558942 |
| 3 | 1250 | Oral-Nasal Mouth Breather | 10 | 0.462968094 | 3.055589422 |
| 3 | 1250 | Oral-Nasal Mouth Breather | 1000 | 46.29680943 | 305.5589422 |
| 3 | 1250 | Oral-Nasal Mouth Breather | 10000 | 462.9680943 | 3055.589422 |

Table 2: Estimated TB exposures from refined modelling and corresponding dosing concentrations b*ased on applying 50µL per well.*

It is of note that, although this method refines the MPPD v3.04 predictions, it predicts a higher local concentration. This results from the following differences: original calculations are performed for total deposited amount in each lung region, and the resulting dose is assumed to be universally deposited over that lung area. This can lead to an underprediction of the local concentration within the lung generations. An example would be a hypothetical exposure that was totally deposited in the trachea. Dividing over the whole TB area will result in a lower concentration than if one only takes the tracheal area, which is done in the case of this refinement. Moreover, the inclusion of TB clearance means that material deposited in lower regions of the lungs will invariably be transported to upper regions, again potentially increasing the local concentration. Hence a total distribution evenly over all surfaces area of a region of the lungs is not necessarily conservative but could in fact underpredict local exposures. Another difference occurs when calculations areng done for a nasal breather only, because the modelling of the local clearance rates is only valid for the trachea and below so clearance rates in the nasal cavity cannot be accounted for here. Hence, the TB deposition is higher.

**Outcome**

Using the MPPD model, with refinement, though still with many associated issues and therefore high uncertainty, TB tissue dosing concentrations spanning 00.009-568.02 pg per cm^2^ would be concluded to replicate those occurring from 8h exposure to inhalable concentrations ranging from 1-10,000 ng per m^3^ varying with activity level, breathing mode and particle sizeMost relevant to an 8h occupational exposure scenario would likely be an adult undertaking light activity (tidal volume of 1250mL) and oral-nasal augmented breathing which returned TB tissues doses of 0.03-278.34 pg per cm^2^ and 0.02-174.44 pg per cm^2^ for particles sizes of 1 and 3µM respectively. Taking a tissue dose range of 0.03-278.34 pg per cm^2^, this would equate to a dosing concentration^[[1]](#footnote-2)^ range of ~0.2-1837 pg per mL (0.0002-2 ng per mL).

**References**

ICRP, Human Respiratory Tract Model for Radiological Protection, Publication 66, Oxford, 1994.

Man, R. et al. (2022). Impact of water uptake and mixing state on submicron particle deposition in the human respiratory tract based on explicit hygroscopicity measurements at HRT-like conditions. Atmospheric Chemistry and Physics, 22, 12387–12399.

1. Concentration of protease needed to deliver the required dose to a 0.33cm^2^ well in 50µl. [↑](#footnote-ref-2)
